# High Calcium Bioglass Enhances Differentiation and Survival of Endothelial Progenitor Cells, Inducing Early Vascularization in Critical Size Bone Defects

**DOI:** 10.1371/journal.pone.0079058

**Published:** 2013-11-14

**Authors:** Karam Eldesoqi, Caroline Seebach, Christina Nguyen Ngoc, Simon Meier, Christoph Nau, Alexander Schaible, Ingo Marzi, Dirk Henrich

**Affiliations:** Department of Trauma-, Hand- and Reconstructive Surgery, Hospital of the Goethe- University, Frankfurt/Main, Germany; Bascom Palmer Eye Institute, University of Miami School of Medicine;, United States of America

## Abstract

Early vascularization is a prerequisite for successful bone healing and endothelial progenitor cells (EPC), seeded on appropriate biomaterials, can improve vascularization. The type of biomaterial influences EPC function with bioglass evoking a vascularizing response. In this study the influence of a composite biomaterial based on polylactic acid (PLA) and either 20 or 40% bioglass, BG20 and BG40, respectively, on the differentiation and survival of EPCs *in vitro* was investigated. Subsequently, the effect of the composite material on early vascularization in a rat calvarial critical size defect model with or without EPCs was evaluated. Human EPCs were cultured with β-TCP, PLA, BG20 or BG40, and seeding efficacy, cell viability, cell morphology and apoptosis were analysed *in vitro*. BG40 released the most calcium, and improved endothelial differentiation and vitality best. This effect was mimicked by adding an equivalent amount of calcium to the medium and was diminished in the presence of the calcium chelator, EGTA. To analyze the effect of BG40 and EPCs *in vivo*, a 6-mm diameter critical size calvarial defect was created in rats (n = 12). Controls (n = 6) received BG40 and the treatment group (n = 6) received BG40 seeded with 5×10^5^ rat EPCs. Vascularization after 1 week was significantly improved when EPCs were seeded onto BG40, compared to implanting BG40 alone. This indicates that Ca^2+^ release improves EPC differentiation and is useful for enhanced early vascularization in critical size bone defects.

## Introduction

Biomaterial-based strategies in bone tissue engineering combine principles of biology and engineering to develop functional substitutes for damaged bone and improve bone regeneration. Designing and fabricating composite biomaterials for bone regeneration from different synthetic biodegradable polymers and bioactive materials is an essential step in engineering bone tissue [1]–[5].

Biomaterials serve as matrices for tissue formation and surface properties promoting cell adhesion, proliferation and differentiation, desirable mechanical properties, non-cytotoxicity and osteoconductivity are all essential [6], [7]. Bioglass (BG), a calcium silicate (CaO-SiO_2_), is similar to the natural inorganic bone component and has been shown to stimulate the formation, precipitation and deposition of calcium phosphates from physiological solution and can result in enhanced bone-matrix interface strength [8].

Recently, composite biomaterials comprising a bioactive bioglass and a biodegradable polymeric component have been developed for bone tissue engineering scaffolds [9], [10]. The composite materials were designed to mimic bone-forming components, to elicit specific cellular responses due to the release of highly reactive components and provide an ideal environment for bone formation [11], [12].

Reparative cells are also an important aspect of bone tissue engineering and they require a biomaterial scaffold, which positively influences cell adhesion, morphology, proliferation and differentiation of neighbouring cells [13], [14]. Relatively little is known about the effect of composite materials containing a bioglass fraction on cells with regenerative capabilities. Previous studies have predominantly focussed on marrow stromal cells (MSC). It has been demonstrated that bioglass materials are well tolerated by MSC, improving their function and differentiation, probably due to calcium ion release [15], [16].

However, depending on the size of the bone defect, the survival and growth of MSC seeded onto a biomaterial influence the in-growth of endogenous bone-forming cells and may be limited due to lacking vascularization and insufficient nutritional bone graft support. Thus, early vascularization of the composite material in the bone defect is a crucial step for in-growth of osteogenic reparative cells in regenerating bone *in vivo* [17]. Improvement of early vascularization has been achieved through the use of endothelial progenitor cells (EPC) [18], [19], isolated from diverse starting populations [20].

In the current investigation “endothelial-like cells” or “early EPCs” were used. These cells are presumably derived from monocytic/dendritic cells coexpressing some endothelial markers together with leukocyte markers and demonstrating a high VEGF synthesis [21]–[24]. In the following ‘early EPCs’ will be referred to as EPCs.

Early EPCs are presumably derived from monocytic/dendritic precursors, express the common leukocyte marker CD45 and some investigators therefore designate them as endothelial like differentiated PBMCs. These cells can be generated in a sufficient amount within 3 to 5 days from a reasonable volume of blood. Early EPCs are potent producers of vascular endothelial growth factor (VEGF) [21]–[24].

Outgrowth EPCs or late EPCs are characterized by a broad spectrum of endothelial markers including VEGF-R2 and UEA-I-Lectin. They express CD34, lack myeloid markers (CD45) and can be expanded *in vitro*. It is likely that these cells are generated from bone-marrow derived CD133+ cells [25], [26]. The culture period of late EPCs is much longer, compared to that of early EPCs. Single colonies of late EPCs appear after 3–4 weeks [27], whereas early EPCs require only 3–5 days [28], [29].

There is wide spread evidence that EPCs can be cultured to be functionally active on ceramic and natural biomaterials, alone and in combination with marrow stromal cells (MSC) [19], [30], [31]. It has also been demonstrated that EPCs seeded on ceramic biomaterials are capable of increasing the vascularization in bone defects within one week after implantation [19], [32], [33].

Recently, the combination of “bone marrow-derived EPCs” which require a culture period of at least 12 days, with polylactic acid (PLA) and a bioglass was analyzed *in vitro* [34]. This composite material promoted EPC mobilisation, differentiation and angiogenesis, likewise probably due to ionic calcium and mechanical cues [34]. No data is available about the effect of PLA-bioglass biomaterial combined with “early EPCs” on early vascularization in a critical size bone defect *in vivo*.

Therefore, the current investigation was designed to elucidate the effect of a composite material consisting of polylactic acid and increasing fractions of bioglass on early EPCs *in vitro.* The amount of calcium released from the composite material was measured and morphological changes and apoptosis of EPCs *in vitro* were recorded. Subsequently, EPCs were incubated with equivalent calcium concentrations and morphological changes, vitality and apoptosis of the EPCs were recorded. The effect of the composite material combined with EPCs on early vascularization (after 1 week) *in vivo* in a critical size calvarian defect model of the rat was also investigated.

## Materials and Methods

### Ethics

Human early EPCs were isolated from buffy coat (Red Cross Blood Donor Service, Frankfurt, Germany). The use of anonymous buffy coat for research purposes was approved by the local ethics committee (Ethik-Kommission des Fachbereichs Medizin der Johann Wolfgang Goethe-Universität, Project No. 329/10) and the donors signed informed consent.

All animal experiments were approved and performed in accordance with regulations set forth by our institution’s animal care and oversight committee located at the Regierungspräsidium Darmstadt (Regierungspräsidium Darmstadt – Veterinärdezernat - Tierschutzkommission, Darmstadt, Germany, Project No. F3/22), in accordance with German law. All surgery was performed under general anesthesia, which was administered intraperitoneally as a mixture of Ketavet and Rompun. All efforts were made to minimize suffering. The animals were sacrificed with an overdose of pentobarbital (150 mg/kg i.p.).

### Biomaterial Characterisation: PLA, BG20, BG40 and β-TCP

**Figure 1:**
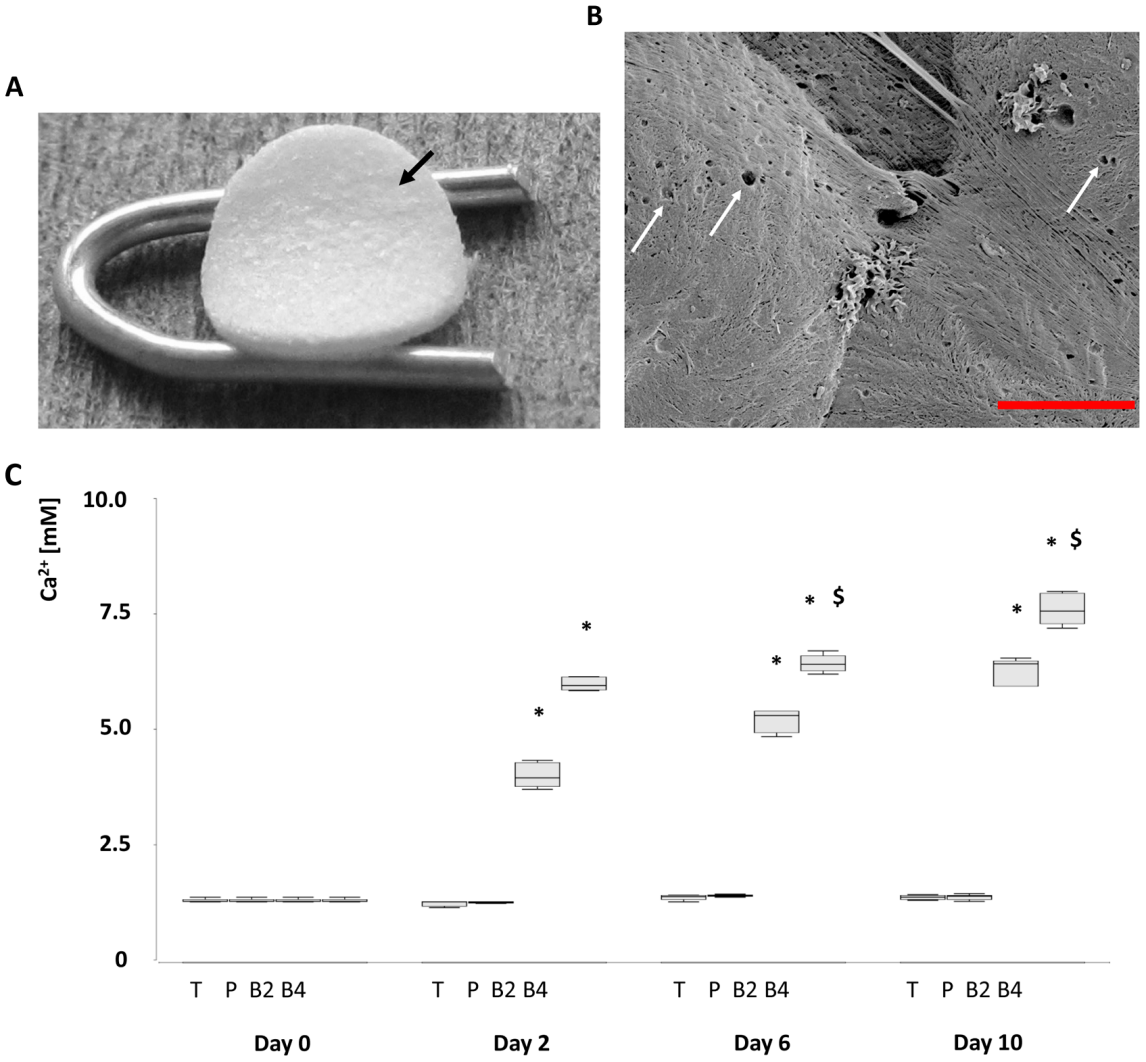
Surface structure and calcium release of the composite scaffold BG40. Disc shaped polymer/bioglass scaffold BG40, with a diameter of 5 mm and a thickness of 1 mm (A). A representative SEM image of BG40 is shown in (B). The composite demonstrates a relatively smooth surface with a thin fibrous texture and small pores (arrows). Scale bar indicates 6 µm. The release of ionic calcium to medium by BG40 (B4), BG20 (B2), PLA (P) and β-TCP (T) is shown in C. The most ionic calcium was released within the first two days. * = p<0.05 BG20, BG40 vs b-TCP, PLA; $ = p<0.05 BG40 vs BG20, n = 5.

The composite biomaterials consisted of a PLA-component supplemented with either 20 or 40% bioglass (BG). Tetraethyl orthosilicate (TEOS, ≥99%) and nitric acid 65% were supplied by Merck Chemicals KgaA (Darmstadt, Germany). Calcium nitrate [Ca (NO_3_)_2_ 4H_2_O, ≥99%], poly (L-lactide) and chloroform (CHCl_3_, ≥99.4%) were purchased from Sigma-Aldrich (Steinheim, Germany). All chemicals were reagent grade and used without further purification. For bioglass synthesis (CaO-SiO_2_ - SiO_2_ 80 mol-%, CaO 20 mol-%), a low viscosity gel was obtained by mixing 31 mL of tetraethyl orthosilicate (TEOS) and 8.6 g of Ca(NO_3_)_2_.4H_2_O in a solution of 5.5 mL of HNO_3_ 2 M, used as catalyst, in 31.5 mL H_2_O. The initial pH was 0.5. The bioglass was cast at room temperature in a Teflon container (Thermo Scientific Nalgene, Germany) until the gel had hardened. Aging was performed at 60°C for 3 days. Drying was carried out at 120°C. The glass was collected in a porcelain crucible (Haldenwanger GmbH, Waldkraiburg, Germany) and heated in a muffle furnace (Nabertherm GmbH, Lilienthal, Germany) at a rate of 3°C/1 minute to 700 C° and maintained at 700°C for 3 hours. The glass particles were ground to powder in a small porcelain mortar (Haldenwanger GmbH, Waldkraiburg, Germany). The particles were then sieved to achieve a size ranging from 106 µm to 125 µm by sieves with a mesh size of 106 µm and 125 µm (Retsch GmbH, Haan, Germany). Composite biomaterials were prepared by mixing polymer [poly(L-lactide)(PLA)] and bioglass with 10 ml chloroform as follows: PLA, PLA/BG 20% and PLA/BG 40% biomaterial. The bioglass content was 0%, 20% and 40% by weight. These biomaterials will be referred to as PLA, BG20 and BG40, respectively. Disc shaped specimens with a diameter of 5 mm and a thickness of 1 mm were cut and stored at room temperature under sterile conditions until use (Fig. 1A). The synthetic β-tricalcium phosphate (β-TCP) Chronos® (Synthes, Dübendorf, Switzerland) was employed as a reference material [19], [31], [32], [33]. The β-TCP particles have a size of 0.7–1.4 mm, porosity 60%, pore size 100–500 mm, low mechanical stability and moderate biodegradability.

### Scanning Electron Microscopy

Surface topography, roughness and morphology of the biomaterial, as well as adherent EPCs, were assessed by scanning electron microscopy (SEM). Untreated biomaterials and biomaterials seeded with EPCs were fixed 5 days after EPC seeding with glutaraldehyde for 30 min and subsequently dehydrated by 15 min incubation in a 5-step ethanol gradient (25%, 50%, 75%, 96%, 100% ethanol). Biomaterials were then incubated overnight in 1,1,1,3,3,3-hexamethyldisilazane (Merck-Schuchardt, Hohenbrunn, Germany) and drained. Afterwards the samples were sputtered with gold (3×60 s, Agar Sputter Coater, Agar Scientific Ltd., UK) using a Hitachi FE-SEM S4500 (Hitachi, Dusseldorf, Germany) with a voltage of 5 kV. The images were digitally recorded using the Digital Image Processing System 2.6 (Point Electronic, Halle, Germany). The public domain software *ImageJ* (http://rsb.info.nih.gov/ij/) was used to evaluate pore size, pore density and fiber diameter.

### Calcium Release

30 mg of cell free BG20, BG40, control PLA or β-TCP were placed in individual wells of 24 well plates and incubated over a period of 2, 6 or 10 days with 1 mL endothelial basal medium (EBM, Cambrex, Verviers, Belgium) supplemented with endothelial growth medium-2MV SingleQuot (Lonza, Basel, Switzerland). At the indicated time points Ca^2+^ in the medium was measured (ABL 800 Flex; Radiometer GmbH, Willich, Germany). The calcium content of untreated EBM-medium served as control.

### Cell Preparation and Culture

#### Isolation of early EPCs from buffy coat and rat spleen

Due to accessibility, early human EPCs were employed for all *in vitro* experiments. To avoid host versus graft reactions EPCs isolated from rat spleen were used in the animal study.

Buffy coat was subjected to density gradient centrifugation (30 min, 900 g) with Ficoll (1.077 g/ml, Biochrom, Berlin, Germany). Recovered mononucleated cells were washed twice with cold PBS^w/o^ (10 min, 900 g) and 2×10^6^ cells/cm^2^ were cultivated on fibronectin coated (10 µg/ml, Sigma, Deisenhofen, Germany) culture dishes with endothelial basal medium (EBM, Cambrex, Verviers, Belgium) supplemented with EGM SingleQuot at 37°C, 5% CO_2_. After 48 hours, non- and weakly- adherent cells were removed, the medium was changed and the cells were cultivated for an additional 72 hrs. A parallel preparation was performed to evaluate the percentage of endothelial differentiated cells. EPCs were identified by staining with 1,1′-dioctadecyl-3,3,3′,3′-tetramethylindo-carbocyanine-labeled acetylated low density lipoprotein (DiLDL, Cell-Systems, St. Katharinen, Germany) in EBM supplemented with 20% FCS. Cells were fixed with 2% paraformaldehyde for 10 min and after washing with PBS^+/+^ FITC-labeled Ulex europaeus agglutinin-1 [10 µg/ml] (lectin, Sigma, Deisenhofen, Germany) were incubated for 1 h. Cells with double-positive fluorescence were considered to be EPCs [28], [29]. Only preparations with a percentage of endothelial like differentiated cells greater than 80% were used. For experiments the cells were detached by accutase treatment (10 min) (PAA-laboratories, Linz, Austria), washed once with EBM+supplements (Cell-Systems, St. Katharinen, Germany) and subsequently adjusted to a density of 5×10^5^ cells in 50 µl. Rat EPCs were isolated from the spleen of homozygous male SD-rats. The spleen was cut in small pieces (approximately 3 mm) and gently mashed using syringe plungers. The cell suspension was filtered through a 100 µm mesh, washed once with PBS and layered onto a ficoll density gradient. The subsequent following isolation, culture and identification were identical to the procedures described for human EPCs.

### Biomaterial Biocompatibility Assessment

#### Seeding efficacy of early EPCs on composite biomaterial

Composite biomaterial chips (BG20, BG40) and PLA chips (Fig. 1a) were individually placed in single wells of a 96-well plate (Nunc, Wiesbaden, Germany) using sterile forceps. Granules of β-TCP were placed in a dense monolayer in single wells of a 96-well plate as additional controls.

5×10^4^ EPCs in a volume of 50 µl were dripped onto the biomaterials and incubated for 10 min at 37°C. After incubation the medium containing non-adherent cells was removed and dripped once again over the biomaterials, followed by incubation as indicated above. This procedure was repeated three times. The bioglass chips or β-TCP granules were then gently transferred to another well containing 100 µl *EBM2*+*EGM2 Singlequot*. The remaining cells in the supernatant and at the bottom of the initial seeding well were isolated, counted and the percentage of adherent cells was calculated ((initial cell number - remaining cell number)/initial cell number)*100. The experiment was performed in duplicate and was repeated 5 times.

### Effect of Composite Biomaterials on EPC Vitality, Morphology, Apoptosis and Gene Expression

Twenty-four well plates were coated with fibronectin as described above. 1×10^5^ EPCs in 1 mL EBM2+EGM2SQ were placed in each well. Transwell inserts with a pore size of 8 µm, containing equal amounts of biomaterials [30 mg of BG20, BG40, control PLA or β-TCP], were placed in the wells. The pores allowed diffusion of soluble biomaterial components to the cell compartment. After 2, 5 and 10 days the morphology of the cells was assessed by phase contrast microscopy at 100 fold magnification. Cells were photographed and the length of the EPCs was evaluated using the software Axiovison 4.7 (Zeiss). An increase in cell length is a cellular characteristic of early EPCs [21], [35].

The funcionality of the cells was assessed by DiL-ac-LDL staining on day 5. The amount of DiL-ac-LDL uptake reflects EPC metabolic status and can be measured by image analysis as mean pixel brightness/cell [19], [30]. DiL-ac-LDL-stained cells were photographed at 100 fold magnification with equal exposure (200 mS]. In each group (control, calcium [10 mM], BG40, BG40+ EGTA [3 mM]) the mean pixel brightness of 20 cells was analyzed (ImageJ (http://rsbweb.nih.gov/ij/).

#### Detection of necrotic EPCs on the biomaterials

Necrotic EPCs were detected by staining nuclei with DAPI (2-(4-Amidinophenyl)-6-indolecarbamidine, Sigma-Aldrich, Deisenhofen, Germany). DAPI passes less efficiently through the membrane of living cells and thus the staining of living cells is much lower [36].

Each 5×10^4^ DiL-ac-LDL prestained EPCs were then seeded onto chips of PLA, BG20 and BG40 that were placed in individual wells of 24-well plates. The seeding procedure was performed as described above. The medium was removed on day 3 and day 5 and a DAPI-solution [final concentration 1 µg/ml in PBS] was added to each well. The DAPI solution was removed after 3 min followed by three washings with 1 mL PBS for each wash. The samples were then subjected to fluorescence microscopy. Five random high power fields were recorded per scaffold at 100 fold magnification. Cells with red fluorescence (DiLac-LDL) were judged as vital cells, cells with red and blue (DAPI) fluorescence were deemed necrotic. Results are presented as number of adhering cells per microscopic field of view (FOV) at 100 fold magnification as well as percentage of necrotic cells per FOV.

To evaluate the effect of Ca^2+^-ions on EPC function, CaCl_2_ was added to EPC cultured in fibronectin coated 24-well plates in the a concentration measured in the aforementioned experiments [10 mM].

Morphological changes and viability of the EPCs were assessed on day 5 after adding CaCl_2,_ as previously described. Additionally, EPC apoptosis was assessed on day 3 after CaCl_2_ addition. The cells were detached by *Accutase* treatment and apoptosis was assessed by means of Annexin-V-staining (Annexin V apoptosis detection Kit, BD-Biosciences, Heidelberg, Germany) and flow cytometry (FACScalibur, BD-Biosciences), according to the manufacturer’s instructions. All experiments described in this paragraph were performed with 5 different EPC-preparations.

#### Expression of endothelial marker genes by Real time RT-PCR

5×10^4^ EPCs were seeded onto PLA, BG20 or BG40 chips and incubated for 3 or 5 subsequent days. EPCs subjected to RNA-isolation on the day of seeding served as day zero.

Total RNA was isolated using the *RNeasy*-system (Qiagen, Hilden, Germany) following the manufacturer’s instructions, with the following exception. Each chip of biomaterial (PLA, BG20, BG40) was individually incubated with approximately 100 µl *RLT* buffer for 3 min, the mixture was gently vortexed and the supernatant was subjected to the RNA isolation procedure. The quality and quantity of RNA was determined using a *NanoDrop* ND-1000 device (Nanodrop technologies, Wilmington, Delaware, USA). Contaminating genomic DNA was removed by digestion with the *RNase free DNase-Kit* following the manufacturer’s protocol (Qiagen).

75 ng of RNA was reverse transcribed using an *Affinity script QPCR-cDNA synthesis kit* (Stratagene, La Jolla, CA, USA), following the manufacturer’s instructions.

Real time RT-PCR was performed on a *Stratagene MX3005p qPCR system* (Stratagene, La Jolla, CA, USA). PCR was performed using the primer assays for human vascular endothelial growth factor (VEGF, NM_003376.4, catalog number PPH00251B) and von Willebrandt factor (vWF, NM_000552.3, catalog number PPH02567E), all purchased from Biomol (SuperArray, Frederick, MD, USA). The expression of glyceraldehyde-3-phosphate dehydrogenase (GAPDH, NM_002046.3, catalogue number PPH00150E) was measured as reference gene.

A melting curve analysis was applied to ensure the specificity of the PCR reaction. Relative quantification of the mRNA levels of the target genes was determined using the comparative CT (threshold cycle values) method (2^−ΔCT^ method). The results are presented as fold change to GAPDH gene expression. This experiment was performed with 4 independent EPC preparations.

### Animals and Cell Transplantation

Eight-week old male Sprague-Dawley rats (n = 12, Charles River, Germany), weighing approximately 350–400 g were housed, four animals per cage under standardized conditions: 15–21°C, air flow and light (12 h day/12 h night), rat food and water ad libitum.

The rats were randomly allocated to the control (n = 6) or experimental group (n = 6). Control animals were implanted with BG40 biomaterial (disc shaped, 5 mm diameter, 1 mm thickness), experimental animals received BG40 seeded with 5×10^5^ rat EPCs 1 h before implantation. General anesthesia was administered intraperitoneally as a mixture of Ketavet and Rompun. All efforts were made to minimize suffering. To create a critical size defect (CSD) the head was shaved and cleaned with antiseptic fluid. A lateral longitudinal incision over the head was made under aseptic conditions. The skull cortex was drilled using a 6 mm bit so that a circular critical calvarial bone defect of 6 mm was created. The biomaterials were implanted into the defect zone and their position controlled. The wound was then closed with continuous subcutaneous stiches using a 4/0-monofilament nylon suture. Animals had free access to food and water and were monitored daily in the postoperative period for any complications or abnormal behaviour.

The animals were sacrificed with an overdose of pentobarbital (150 mg/kg i.p.) and weighed after 1 week. The skull bone was dissected free and removed. Bones were wrapped in gauze moistened with physiologic NaCl-solution and stored at −80°C until preparation for immuno-histological examination.

### von Willebrand Factor (vWF) as Marker of Early Vascularization

Skull bones were fixed in 4% *Zinc-Formal-Fixx, 4%* (Thermo Electron, Pittsburgh, USA) for 20 hrs, decalcified over 7 days in a 10% Tris buffered EDTA-solution under continuous stirring and embedded in paraffin. Sections (5 µm) of the decalcified specimens parallel to the long axis of the head were stained with hematoxylin and eosin or incubated with a mouse anti vWF-antibody, which crossreacts with both human and rat vWF (8 µg/mL, Biomol, Hamburg; Germany). An isotype identical (IgG_1_) non specific mouse antibody served as negative control (Dako, Hamburg, Germany). As secondary antibody, a polymer HRP-conjugated goat anti mouse antibody (Histofine simple stain rat Max Po (M), Nichirei Biosciences, Tokyo, Japan) was applied and the sections were incubated with 3-amino-9-ethylcarbazole (AEC, Lab Vision, Dreieich, Germany). Finally, a counterstain with hematoxylin was performed. One slide per animal was analyzed using light microscopy (Axioobserver Z1, Zeiss, Gottingen, Germany) at a magnification of 100x in combination with a computer-supported imaging picture analysis system (Axiovision 4.7; Zeiss). Von Willebrandt factor positive blood vessels with a lumen were counted in 6 non overlapping images/slide/animal sourrounding the defect area and the mean number of vessels was calculated. These means were subsequently used for statistical anlysis. Cells positive for von Willebrandt factor were not considered. Imaging and blood vessel counting were performed with blinded specimens examined in random order by an independent observer, blinded to the group setup.

### Statistics

Results are presented as box-plots of the median in figures, 25% and 75% quartiles ((M (25%q/75%q)) in the text and tables. Nonparametric Kruskal–Wallis test and multiple Conover-Iman test were consequently used, and a Bonferroni-Holm corrected p<0.05 was used to indicate statistical significance. Statistics were calculated using the software *Bias* 10.03 (Epsilon-Verlag, Darmstadt, Germany).

## Results

### Biomaterial Characterization and Calcium Release

Scanning electron microscopy revealed a relatively smooth surface of the composite biomaterial BG40. Higher magnification revealed fibrous structure with a fiber diameter of approximately 0.1 µm. The fibres were partly arranged in parallel structures but also significant areas of irregularly arranged fibres were found. Putatively not interconnecting micropores with a diameter ranging from 0.2 µm up to 1.2 µm were evenly distributed on the surface at a median density of 58 micropores/1000 µm^2^ (Figure 1B).

The release of ionic calcium by BG20 as well as BG40 was significantly elevated in comparison to β-TCP during the whole 10 day observation period. The amount of released calcium ions through BG40 was significantly higher, compared to BG20 (d6, d10). The majority of calcium was released between days 1 and 2 (BG20 57% of total d10 release); BG40 74% of total d10 release, Figure 1C).

### Seeding Efficacy of Early EPCs on the Composite Biomaterial

**Figure 2:**
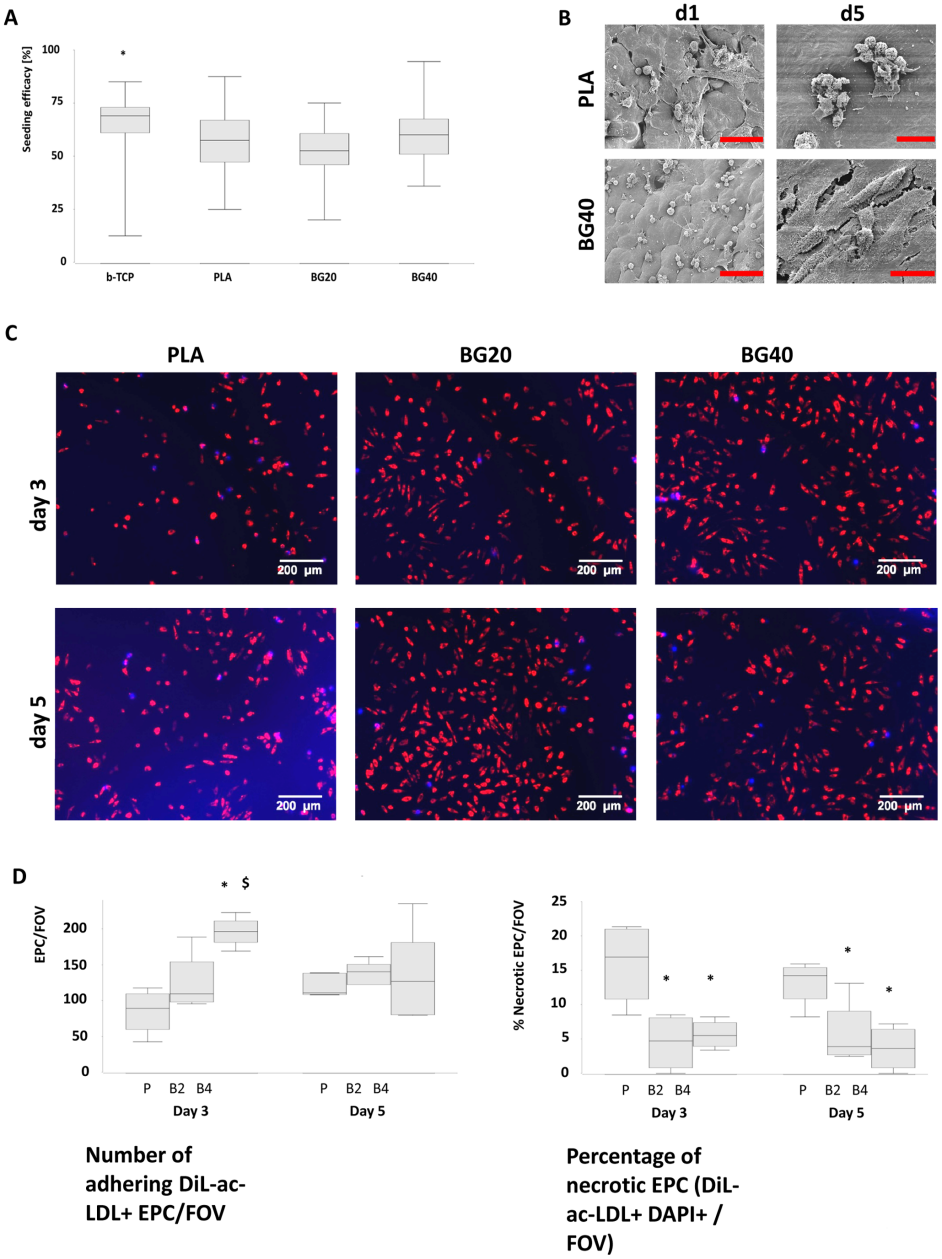
Seeding efficacy and EPC phenotype on the composite scaffold. Seeding efficacy of EPCs on BG40, BG20, PLA and β-TCP (A). The percentage of initially adhering EPCs on β-TCP was significantly increased compared to PLA. Appearance of EPCs seeded on BG40 on day 1 and day 5 (B). Representative SEM images are shown. EPCs demonstrated a spherical structure on the first day after seeding on BG40 whereas a flattened appearance was seen when EPCs were seeded on PLA. On day 5 EPCs presented a necrotic phenotype when seeded on PLA, whereas they appear vital on BG40. Scale bars indicate 60 µm (day 1: PLA, BG40) respectively 20 µm (day 5: PLA, BG40). *: p = 0.045 β-TCP vs. PLA, n = 5. Confirmation of increased necrosis of EPC on PLA compared to BG20 and BG40, representative fluorescence microscopy images were shown in (C) at an original magnification of 100 fold. DiL-ac-LDL prestained EPCs were seeded onto the scaffolds. DAPI was added over a period of 3 min to discriminate between live (red cytoplasm, no blue nucleus) and necrotic EPCs (red cytoplasm, blue nucleus) on day 3 or 5 after seeding. The quantitative evaluation of this experiment is depicted in (D). The number of EPC was significantly lowered on PLA on day 3 (p = 0.02 vs BG20, p = 0.01 vs BG40) and the percentage of necrotic EPCs differed significantly between PLA and BG40 on day 3 (p = 0.02) and day 5 (p = 0.02). *: p<0.05 vs. PLA, $: p = 0.045 BG40 vs. BG20; P: PLA; B2: BG20; B4: BG40, n = 5. Scale bar indicates 200 µm.

Seeding efficacies of EPCs were comparable between β-TCP and BG40. The percentage of initially adhering EPCs was significantly decreased on PLA, compared to β-TCP (Figure 2A).

### Vitality and Function of EPCs Cultured on the Composite Biomaterial

Scanning electron microscopy performed on the first day after EPC seeding revealed evenly distributed EPCs on PLA and BG40. EPCs demonstrated a rounded phenotype on BG40 and a more flattened appearance when seeded on PLA (Fig. 2B). EPCs cultured 5 days on PLA demonstrated a necrotic phenotype, whereas EPCs cultured for the same time period on BG40 had a vital appearance (Fig. 2B).

Additional fluorescence microscopy analysis was performed for confirmation. Dil-ac-LDL prestained EPC were seeded onto the biomaterials. The number of adhering EPC was significantly decreased on PLA in comparison to BG20 and BG40 on day 3 after cell seeding but those differences were levelled on day 5, though (Fig. 2C). Necrotic EPC were identified through an additional DAPI-staining as indicated in the Materials and Methods-section. The percentage of necrotic EPC was significantly increased on PLA compared to EPCs that have been cultured on BG20 and BG40 (Fig. 2C, 2D).

The gene expression of VEGF and vWF was evaluated to determine the endothelial function of EPCs cultured on the composite materials over a period of 5 days. Gene expression is presented as fold change of the housekeeping gene expression (GAPDH). VEGF gene expression remained stable on BG40 on day 3 and day 5 in comparison to day 0, whereas VEGF gene expression decreased singificantly from day 0 to day 5 on BG20 and PLA. No significant differences were found between the PLA, BG20 and BG40 on day 3 and day 5 however a trend towards higher values were seen for BG40 vs PLA on day 5 (p = 0.1) (Table 1). The gene expression of vWF increased significantly from day 0 to day 5 for all biomaterials. No significant differences between PLA, BG20 and BG40 were found albeit a trend for increased vWF-gene expression on BG40 in comparison to PLA on day 5 (p = 0.1, Table 1).

**Table 1:** Gene expression of VEGF and vWF in EPC cultured on PLA, BG20 and BG40 over a period of 5 days.

Gene	Biomaterial	Day 0	Day 3	Day 5
**VEGF**	**PLA**	0.0079	0.0026	0.0014
		(0.0045/0.0107)	(0.0016/0.0066)	(0.0002/0.0027)
				↓ p = 0.03 vs. day 0
	**BG20**	0.0079	0.0008	0.0013
		(0.0045/0.0107)	(0.0005/0.002)	(0.0007/0.0256)
			↓ p = 0.05vs. day 0	
	**BG40**	0.0079	0.0425	0.0075
		(0.0045/0.0107)	(0.0004/0.35)	(0.0025/0.014)
				↑ p = 0.1 vs PLA day 5
**vWF**	**PLA**	0.0056	0.0136	0.0153
		(0.0017/0.0072)	(0.0102/0.0184)	(0.0105/0.0211)
				↑ p = 0.04 vs day 0
	**BG20**	0.0056	0.018	0.0141
		(0.0017/0.0072)	(0.0073/0.013)	(0.0111/0.1902)
				↑ p = 0.05 vs day 0
	**BG40**	0.0056	0.266	0.0728
		(0.0017/0.0072)	(0.0233/1.017)	(0.0276/0.1224)
			↑ p = 0.04vs day 0	↑ p = 0.04 vs day 0
				↑ p = 0.1 vs PLA day 5

The values are given as fold change to the expression of the GAPDH gene which served as housekeeping gene (median value (25% quartile/75% quartile). Day 0 demonstrates the gene expression of the EPC before seeding on the biomaterials. Generally, gene epression of VEGF declined over the time whereas gene expression of vWF increased during the observation period. The experiment was performed with 4 independent EPC preparations. ↑ = increased gene expression; ↓ = decreased gene expression.

### Effect of Composite Scaffolds on EPC Morphology, Function and Survival

**Figure 3:**
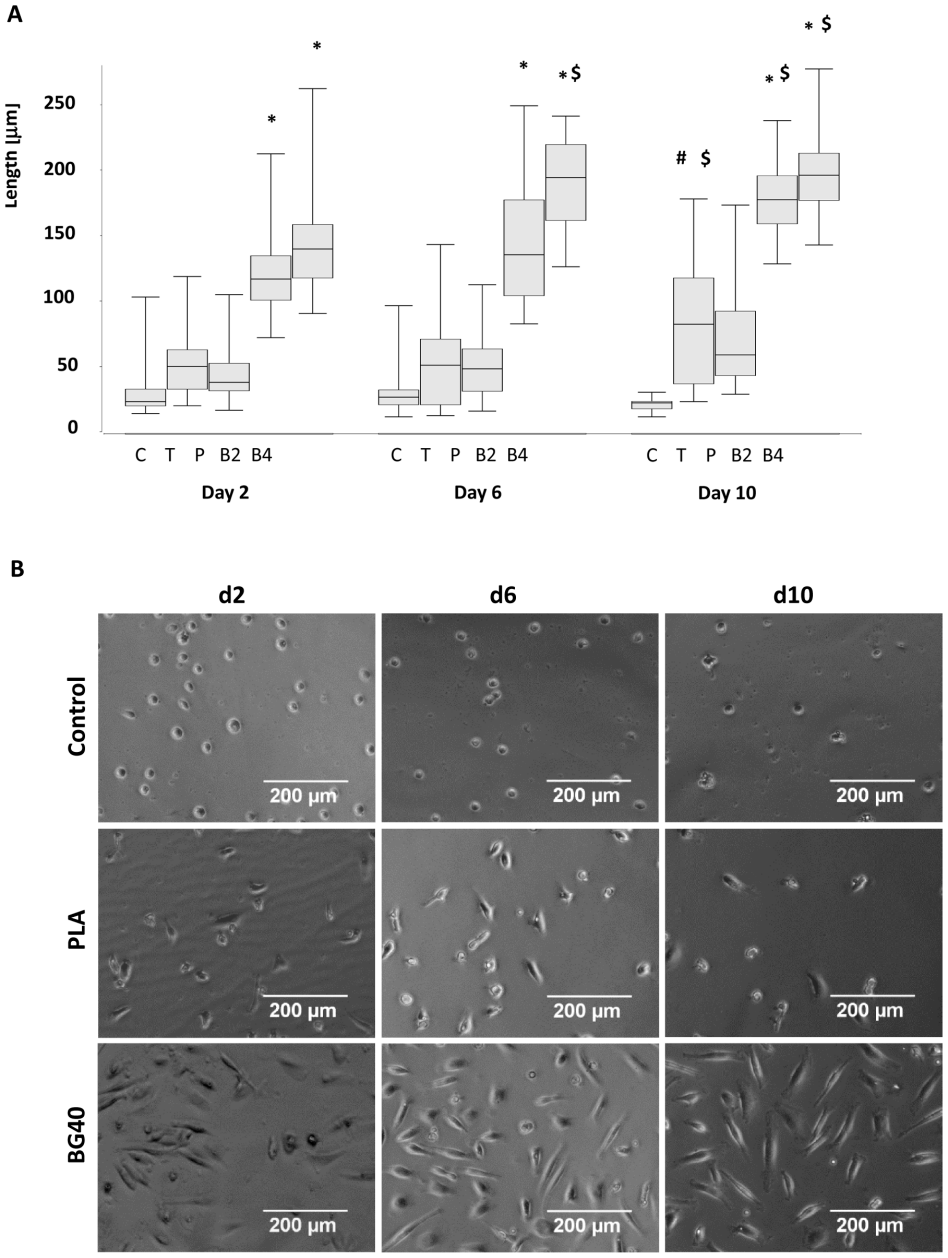
Coincubation of EPCs with BG40 and BG20 leads to EPC elongation. Incubation with BG20 (B2) and BG40 (B4) lead to a significantly sustained increase in EPC length compared to EPCs incubated with β-TCP (T), PLA (P) or the medium control (C) during the whole observation period (A). The length of EPCs incubated with BG20 and BG40 increased significantly from day 2 to day 10 (A). Representative micrographs of EPCs incubated with either medium, PLA or BG40 demonstrated EPC elongation (B). * = p<0.05 BG20, BG40 vs control, β-TCP, PLA; $ = p<0.05 d6, d10 vs d2; # = β-TCP vs control, n = 5.

The co-incubation of EPCs with BG40, and to a lesser extent with BG20, resulted in significant EPC elongation compared to control (medium), PLA and β-TCP. These changes became prominent on day 6 and day 10 after incubation started (Figure 3A, B).

**Figure 4:**
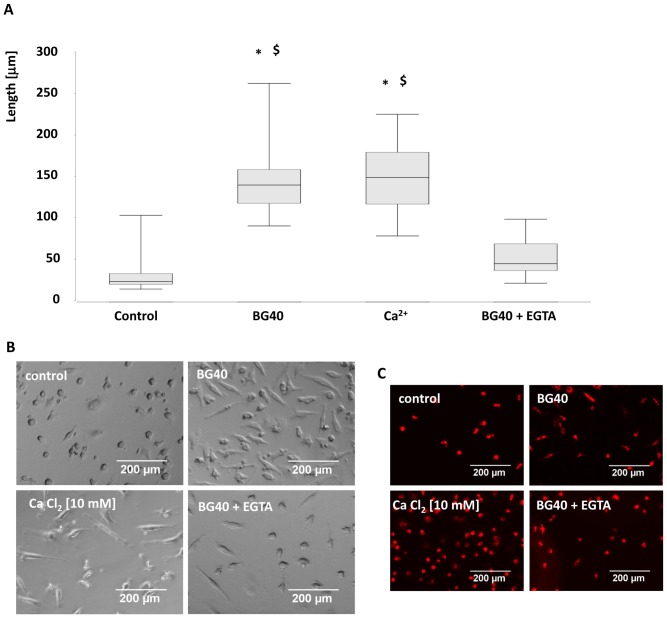
Calcium released by BG40 induces EPC elongation. EPCs were incubated in the presence of either 30/mL, 10 mM Ca^2+^ or BG40 conditioned medium+EGTA [3 mM]. Cell length was determined on day five (A) by means of phase contrast microscopy and subsequent histomorphometric evaluation. In (B) corresponding micrographs of EPCs are shown. The elongation of the EPCs was significantly lower in the presence of the calcium chelator EGTA [3 mM]. The uptake of DiL-ac-LDL demonstrated the endothelial phenotype and EPC vitality. The mean DiL-ac-LDL amount per cells was not significantly altered among the groups (control, 10 mM calcium, BG40, BG40+EGTA) (C). * = p<0.05 vs control; $ = p<0.05 vs BG40+EGTA, n = 5. Scale bars indicate 200 µm.

To test the hypothesis that calcium released by the composite biomaterials is critical, calcium was added to the medium in a final concentration of 10 mM. This concentration was comparable to the calcium released by BG40 in the experimental setting (Figure 1C). The elongation of the cells was assessed five days later. In line with the aforementioned results a statistically significant increase in cell length was observed (Figure 4A, B). As final proof that calcium derived from bioglass induces EPC elongation, EPCs were incubated over a period of five days with medium conditioned with BG40 (over 2 days) in the presence or absence of the specific calcium chelator EGTA (ethylene glycol tetraacetic acid). The addition of EGTA lowered the Ca^2+^ concentration from 10 to approximately 2 mM. A significant decline of EPC length in the presence of EGTA was apparent (Figure 4A). A Dil-ac-LDL-staining performed in parallel proved the vitality and endothelial function of the EPCs (Figure 4A, B, C). The mean DiL-ac-LDL uptake per cell was comparable in all groups (Control: 31 (25/44); 10 mM CaCl_2_∶27 (22/43); BG40∶36 (26/46); BG40+EGTA: 32 (27/44), not significant).

**Figure 5:**
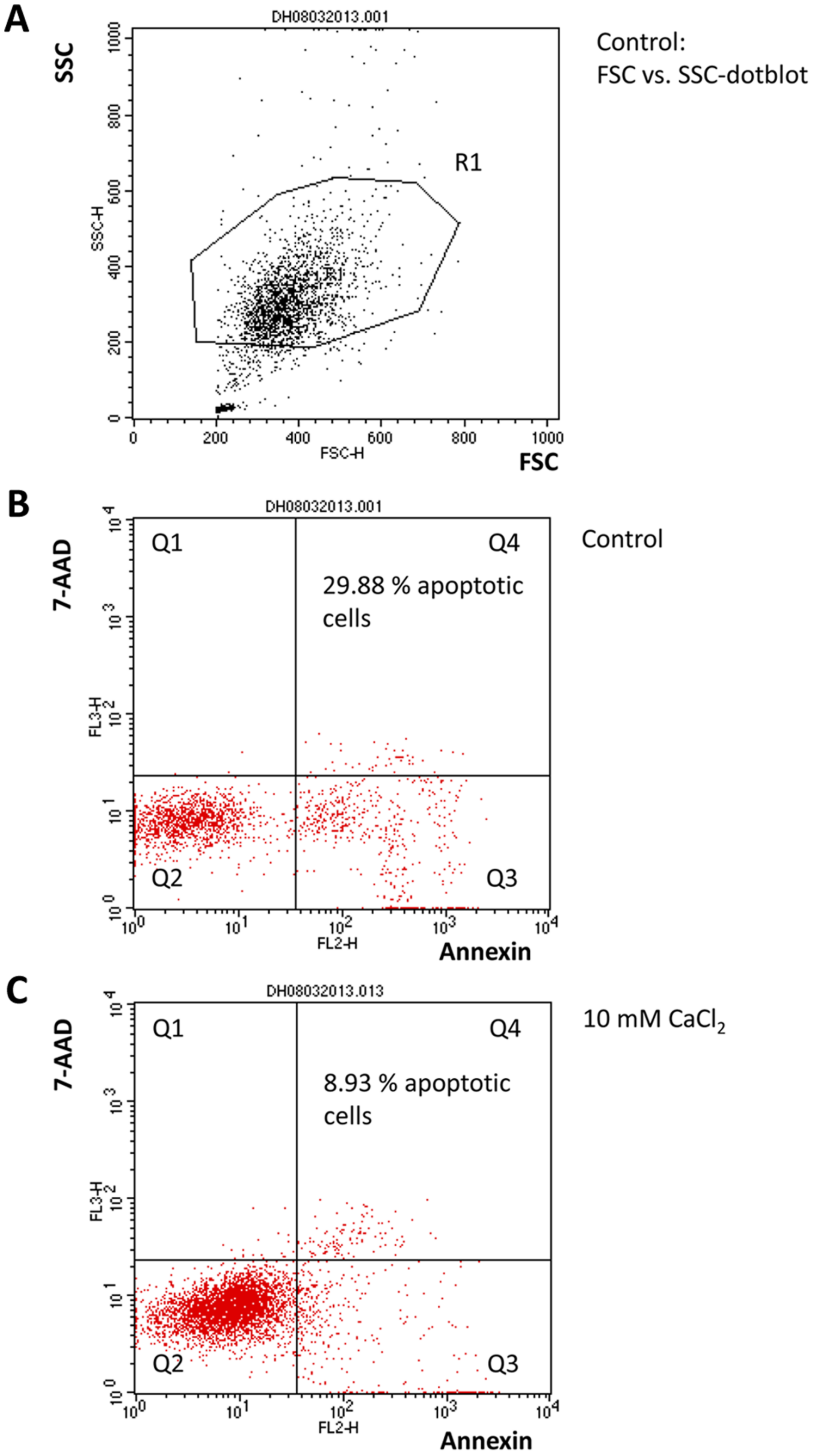
Decline of EPC apoptosis after 3 day incubation with Ca^2+^ [10 mM]. Apoptosis was measured by Annexin-staining, necrotic cells were stained with 7-AAD. Representative dot blots of the FACS-analysis are presented. Cells with appropriate forward/sideward scatter characteristics were gated (“R1”, A). The percentage of apoptotic cells was subsequently determined through quadrant analysis. Early and late apoptotic EPCs were located in quadrants Q3 and Q4, respectively. The percentage of apoptotic cells consists of the sum of early and late apoptotic cells. Apotosis of early EPCs in the control experiment is shown in (B) and in the presence of Ca^2+^ in (C).

Next, the influence of Ca^2+^ ions added to the medium on EPC survival was investigated. In the control experiment the number of EPC/field of view (FOV) declined significantly between day 1 and day 5 (99 (78/130) vs 51 (46/57), p<0.05). In contrast, EPCs/FOV remained stable between days 1 and 5 in the presence of BG40 (Day 1∶106 (93/120) vs day 5∶93 (86/99), not significant) or CaCl_2_ [10 mM, added to medium] (Day 1∶104 (91/111) vs day 5∶85 (75/105), not significant). Additionally, cell numbers in the presence of BG40 or CaCl_2_ in the medium were significantly elevated, compared to the control at day 5. To investigate whether EPC maintenance was due to CaCl_2_ mediated apoptosis inhibition, EPC apoptosis over a period of three days with and without 10 mM CaCl_2_ was followed by apoptosis measurement. A significantly decreased rate of apoptotic EPCs under the influence of 10 mM CaCl_2_, compared to controls was observed (Control: 30.4% (28.1/33.2) vs CaCl_2_∶8.6% (8.5/10.6); p<0.05). Representative dotblots of the measurement are depicted in figure 5.

### Effect of BG40 and BG40+EPC on Early Vascularization in vivo

**Figure 6:**
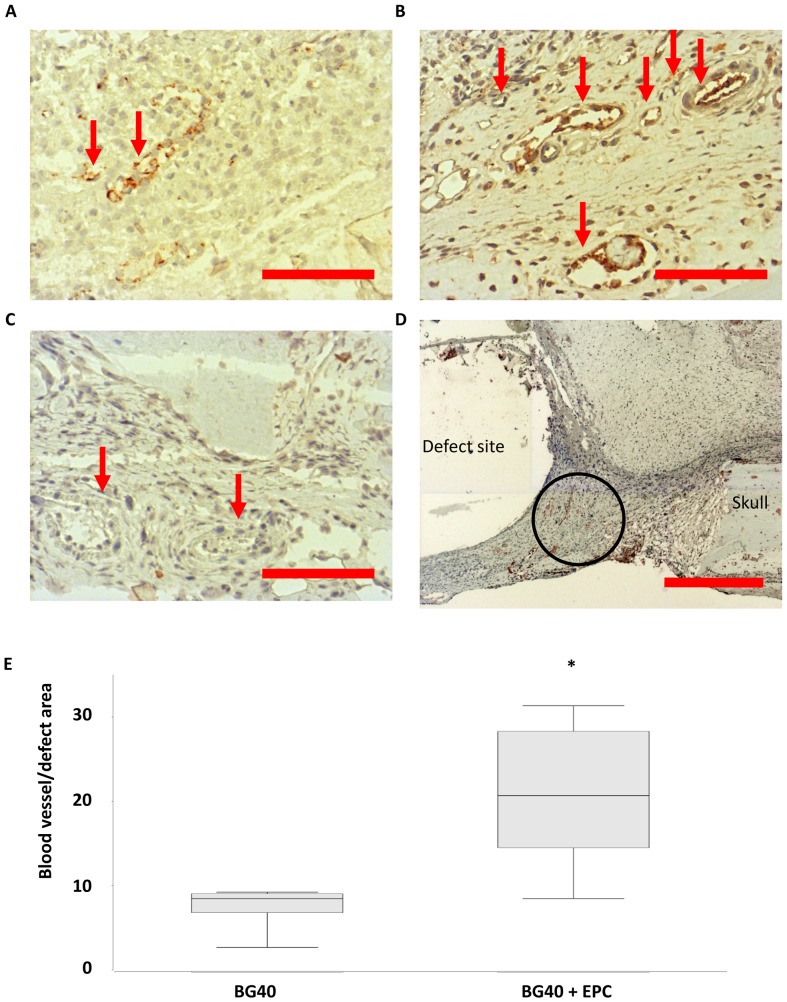
BG40+EPC increase vascularization one week after implantation. Representative vWF-stained histological sections of the defect site of male rats treated with BG40 (A, n = 6) and BG40+EPC (B, n = 6). The blood vessel density increased significantly in animals treated with BG40+EPC, compared to BG40 alone (B). vWF-positive structures appear brown (arrows). The isotype control is presented in figure 6C. The absence of staining in blood vessel structure (arrows) indicates the specificity of the vWF-staining (C). An overview of the defect area is provided in d (original magnification 50x). The BG40 implant could not be preserved during sample sectioning and left a gap corresponding to the BG area (defect site). The skull bone is located in the right corner (skull). The encircled area marks the region shown at higher magnification (original magnification 200x) in (A) and (B). The quantitative evaluation of vWF-positive blood vessel structures is depicted in (e). All vWF-positive, blood vessel like structures in the neighborhood of the whole defect site were counted. Red scale bars: 100 µm (A, B, C), 500 µm (D), *: p = 0.015.

Areas of neovascularization in the critical calvarial bone defect of rats treated with BG40 or BG40+ EPC were detected after 1 week by vWF staining (Fig. 6A, 6B).

The histomorphometric analysis demonstrated significantly higher blood vessel density surrounding the defect area in samples obtained from animals receiving BG40 loaded with EPCs, compared to samples from animals treated with BG 40 alone (Fig. 6E, p<0.015). Besides the increased amount of vWF positive structures, more large vessels in the newly formed tissue were detectable (Fig. 6A, 6B) under the influence of BG40+ EPCs, compared to BG40 alone.

## Discussion

This study demonstrates that EPCs can successfully be cultured on a PLA/bioglass composite biomaterial and that calcium released from the bioglass fraction leads to significant elongation and significantly improved survival of EPCs *in vitro*. *In vivo,* EPCs seeded onto BG40 induce significantly improved early vascularization to a critical size skull defect in the rat one week after implantation.

### Physical Properties

Ideal biomaterials for bone reconstruction should fulfill requirements including mechanical stability, osteoinductivity, osteoconductivity and support of revascularization. Currently available single component materials do not meet all these requirements. Hence, more sophisticated biomaterials are needed and combining different biodegradable biomaterials with complementary properties may circumvent individual shortcomings.

In the present investigation a novel composite biomaterial based on a PLA carrier and a bioglass fraction, consisting of calcium oxide and silicium dioxide, was investigated. PLA is highly biocompatible with better thermal processability than other biopolymers. The main limitations of PLA are poor toughness, slow degradation and hydrophobic properties resulting in low cell affinity [37]. Pure bioglass is hard and brittle but offers a surface suitable for cell attachment. It is highly biodegradable and influences the local environment by releasing bioactive ions such as ionic calcium [38], which may lead to improved cellular responses at the implantation site [39].

The composite BG40 demonstrated a relatively smooth surface with fibrous structures in the submicrometer scale, and small pores were present in high density. The occurrence, size and form of distinct micro- and macroporous structures on PLA−/bioglass based biomaterials depend on the fabrication process [37]. Apart from structural aspects due to different fabrication processes, composites containing a bioglass component share the common feature of releasing ions such as calcium.

In line with previous reports a significant Ca^2+^ release was observed with the bioglass composites employed in this investigation. The vast majority of ionic calcium was set free within the first two days, but further calcium release to the medium occurred over the following eight days. Similar release dynamics have been obtained using PLA with a bioglass fraction [34] and PGLA composites supplemented with bioglass [40]. However, the amount of calcium released has differed among studies, possibly due to the different fractions of bioglass within the biomaterials and/or different amounts of biomaterials used in the calcium release experiments.

It should be noted that the form of the biomaterials influences the properties of the biomaterial [41]. In the current investigation film like discs of the composite material were employed. Granules of BG20 and BG40 might have accelerated calcium release due to the higher surface area compared to disc shaped biomaterials. A greater surface area might also allow higher loading with regenerative cells.

### Bioglass Effect on Cells and Vascularization in vitro and in vivo

The initial adhesion of EPCs on the bioglass composites BG20/BG40 and on β-TCP was comparable but significantly lower, compared to former studies [30]–[32]. These deviating results might be explained by alterations in the seeding procedure, which was performed in 96 well plates with comparatively low cell numbers in the present investigation. In former studies an EPC seeding efficacy of over 90% on β-TCP has been demonstrated [19]. Seeding efficacy on BG20/BG40 may be equally good if the same seeding conditions are applied.

The *in vitro* investigation demonstrated that BG40 induced the best EPC differentiation and survival, which hypothetically could result in better vascularization of a bone critical size defect *in vivo*.

To test this, the composite biomaterial BG40 with or without EPCs, was implanted into a critical size calvarial defect and early vascularization was analyzed. Much better vascularization was observed in animals receiving BG40 and EPCs. The experimental setup did not provide an evaluation of the effect of BG40 alone on early vascularization since BG40 without EPCs was not tested.

Two factors possibly play a dominant role in the increased *in vivo* vascularization. First, it has been demonstrated in different animal models that early EPCs increase vascularization [32], [33], [42]. Second, the beneficial effects of calcium ions released by the bioglass component might also contribute to the increased vascularization in *vivo* by supporting EPC function and survival. Separating the effects of the bioglass from the effects of EPCs on vascularization in this *in vivo* model will require future investigation.

The contribution of early EPCs in forming blood vessels is a matter of debate. Crosby and colleagues have reported that 8.3% –11.2% of endothelial cells that developed in sponge induced granulation tissue over 1 month were derived from circulating hematopoietic progenitor cells [43]. Hence, it is has been proposed that early EPCs more likely act in a paracrine manner, secreting proangiogenic factors such as VEGF [22]. Previous findings, whereby human early EPCs were implanted in a rat critical size defect of the femur, support this hypothesis. When the animals were sacrificed one week after implantation and the VEGF expression and distribution of the human cells were analyzed a significantly elevated expression of VEGF in the defect area, compared to control animals without EPC, was observed. In contrast, the incorporation of the human cells in vWF-positive vessel structures was a rare event [33].

A stimulating effect of ionic products released by bioglass on osteogenic cell types has been reported in several investigations. Kaufmann et al. have reported an up-regulation of osteogenic marker genes such as osteocalcin, osteonectin and osteopontin in osteoblasts cultured with bioglass [44]. Jell and coworkers have reported a significant up-regulation of osteogenic markers such as alkaline phosphatase and bone sialoprotein in osteoblasts incubated with bioglass conditioned medium [45].

More recently, different aspects of the proangiogenic potential of biomaterials containing bioglass have also been described. Day and colleagues have demonstrated enhanced synthesis of VEGF and basic fibroblast growth factor after incubating fibroblasts with bioglass [46]. Leu and Leach have shown that low concentrations of bioglass possess proangiogenic potential when they incubated human microvascular endothelial cells with bioglass conditioned medium. A dose dependent significant increase in cell proliferation, tube formation and VEGF-synthesis *in vitro* was observed [47]. The same group analyzed the angiogenic response to bioactive glass in an irradiated calvarial defect in rats. A bilateral calvarian defect was created and filled with a bioglass supplemented collagen sponge. An empty collagen sponge served as control. Significantly greater neovascularization within the defect in the presence of bioglass indicates a proangiogenic effect of this material [48].

The effect of a composite biomaterial consisting of a PLA-component and bioglass on EPC has recently been investigated by Aguirre et al. [34]. They report a significant increase of VEGF production and tube density if EPCs were seeded on a PLA-bioglass composite. However, the type of EPCs that were used in that study is not clearly defined. Based on the protocol for isolation and differentiation of the EPC it may be assumed that “late” EPCs (CD133 positive cells) were employed.

In the present study early EPCs were used [19], [30]–[33]. In accordance to Aguirre et al. [34] an elongation of EPCs, which indicates sprouting activity [21], [28], [35], was observed after incubation with bioglass, correlating to the calcium concentration available in the medium.

A trend toward increased gene expression of VEGF and vWF was seen after incubation of EPC on BG40 in comparison to EPC cultured on PLA. This result is not as distinct as described by Aguirre et al who observed a significant up regulation of VEGF in EPC after incubation on a PLA-bioglass composite material [34]. This inconsistency might be due to differences in the mixture of the composite biomaterials, the sort of EPC and different measurement points (day 1 [34] vs. day 3, day 5 in this work). Calcium ions released by the bioglass might enhance the expression of both genes as it was discussed for VEGF by Aguirre et al [34]. The general increase of vWF gene expression of early EPC cultured on PLA and PLA-bioglass composite materials has not been described yet and might indicate a generally advanced endothelial differentiation of the EPC over the time [21].

The phenotypic changes of the early EPC were associated with a decline in EPC apoptosis in our experiments. Calcium mediates diverse cell processes and improved EPC survival may be due to a calcium dependent activation of PI3K, which activates the protein kinase Akt, a multifunctional regulator of EPC survival [28]. Cell shape alteration might occur over calcium mediated activation of rho-kinase (ROCK), a key regulator of cytoskeleton and cell polarity [49].

### Conclusion

The present study demonstrates that the composite materials BG20 and BG40 support the function and survival of EPCs *in vitro* and that those effects are mediated by calcium released from the biomaterials. These observations suggest that improved EPC sprouting and delayed apoptosis should not only be seen as a pure *in vitro* phenomenon but may also be relevant to the improved vascularization in the skull defect model *in vivo*. It has become evident that understanding the effects of calcium ions on EPCs *in vitro* and *in vivo* requires further investigation. Elucidating underlying molecular mechanisms and subsequent optimization of the ionic components of bioglass may help to develop more effective biomaterials.
